# Adaptive Diversification in Genes That Regulate Resource Use in Escherichia coli

**DOI:** 10.1371/journal.pgen.0030015

**Published:** 2007-01-19

**Authors:** Christine C Spencer, Melanie Bertrand, Michael Travisano, Michael Doebeli

**Affiliations:** 1 Department of Zoology, University of British Columbia, Vancouver, British Columbia, Canada; 2 Department of Biology and Biochemistry, University of Houston, Houston, Texas, United States of America; 3 Department of Mathematics, University of British Columbia, Vancouver, British Columbia, Canada; University of Toronto, Canada

## Abstract

While there has been much recent focus on the ecological causes of adaptive diversification, we know less about the genetic nature of the trade-offs in resource use that create and maintain stable, diversified ecotypes. Here we show how a regulatory genetic change can contribute to sympatric diversification caused by differential resource use and maintained by negative frequency-dependent selection in Escherichia coli. During adaptation to sequential use of glucose and acetate, these bacteria differentiate into two ecotypes that differ in their growth profiles. The “slow-switcher” exhibits a long lag when switching to growth on acetate after depletion of glucose, whereas the “fast-switcher” exhibits a short switching lag. We show that the short switching time in the fast-switcher is associated with a failure to down-regulate potentially costly acetate metabolism during growth on glucose. While growing on glucose, the fast-switcher expresses *malate synthase A* (*aceB),* a critical gene for acetate metabolism that fails to be properly down-regulated because of a transposon insertion in one of its regulators. Swapping the mutant regulatory allele with the ancestral allele indicated that the transposon is in part responsible for the observed differentiation between ecological types. Our results provide a rare example of a mechanistic integration of diversifying processes at the genetic, physiological, and ecological levels.

## Author Summary

Understanding the origin of diversity is a fundamental problem in evolutionary biology. The past decade has seen a shift in our understanding of speciation, away from considering geographical isolation as the main cause and towards elucidating how ecological interactions can drive diversification in populations that occupy a single and contiguous spatial area, a process called sympatric diversification. By culturing bacteria over many generations it is possible to observe processes of diversification in real time. This paper characterizes diversification caused by ecological interactions in bacteria at the physiological and genetic level. Propagating a single ancestral E. coli strain on a mixture of two resources, we found sympatric diversification into two descendant strains. This diversification occurs in a shared, well-mixed environment and is caused by competition for resources. We show that 1) the diversified strains use physiological pathways differently to consume the resources, 2) this physiological difference is caused by differences in the expression levels of genes controlling metabolism, and 3) this difference in gene expression is influenced by genetic differences in regulatory genes. Our paper thus contributes to an integrative understanding of sympatric diversification in E. coli at the genetic, physiological, and ecological levels.

## Introduction

Adaptive diversification describes the splitting of an ancestral lineage into two derived groups due to frequency-dependent ecological interactions [1]. During this process, disruptive selection on a common ancestral type drives the creation of diversified ecotypes through a series of adaptive genetic changes [1,2]. Even though this process is of central importance in evolutionary biology [3], examples are rare where the genetic changes that differentiate adaptively diversified species or ecotypes are known [4–8]. Even when genetic differentiation can be identified, it is often hard to establish a link to phenotypic differentiation, and even harder to show that the associated phenotypes played a causal role in the ecological mechanisms driving diversification [4–7].

Microbial experimental model systems greatly facilitate our ability to connect genotype to phenotype [9]. For instance, in static microcosms Pseudomonas fluorescens can readily diversify into three morphological types from a common ancestor [10–13]. One of these, the wrinkly spreader, dominates the liquid-air interface by forming a biofilm that it creates by overexpression of a polymer-forming operon [7]. While static microcosms provide different spatial niches and therefore an obvious mechanism for niche differentiation, it is less clear how diversification can occur in homogeneous liquid culture. We have used the bacterium Escherichia coli to investigate the ecological and genetic mechanisms of adaptive diversification in a homogeneous, well-mixed environment.

When E. coli evolve in shaken serial batch culture with daily depletion of a mixture of glucose and acetate, in each batch they first use up all the available glucose and then undergo a diauxic switch to acetate consumption before entering stationary phase, after which they are transferred to a new batch of resources. After 1,000 generations in this homogeneous two-resource environment, E. coli readily diversify into two ecotypes that show different patterns of diauxic resource use. These types were previously dubbed Large and Small to reflect their colony morphology when cultured in a nutrient-rich environment [14–16]. Relative to the Small colonies, Large colonies exhibit high growth rates on glucose, slow growth rates on acetate, and a long lag between growth on glucose and growth on acetate. Thus, the diauxic growth profile of Larges is markedly different from the diauxic growth pattern of Smalls (Figure 1A).

**Figure 1:**
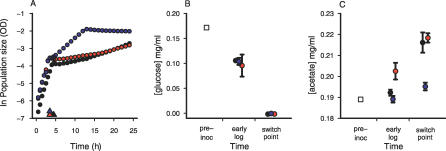
E. coli Growth Profiles of Each Isolated Strain on Glucose/Acetate Mixture and the Change in Glucose and Acetate Concentrations in First Phase of Diauxie (A) Three isolated genotypes (FS, SS, and Anc) consumed glucose (hours 0 to ∼4), then acetate (hours ∼4 until maximum OD), in a standard diauxic (two-stage) growth pattern. The SS (red) closely paralleled the ancestral (black) strain in growth patterns on acetate, while the FS (blue) exhibited a much faster diauxic switch from growth on glucose to growth on acetate and a higher growth rate on acetate (second stage of diauxie). Triangles on the horizontal axis indicate the switching points for each strain. (B) All strains deplete glucose from the medium during the first phase of diauxie (from inoculation into batch culture to the time of diauxic switch). (C) Change of acetate concentration in the medium during the first phase of diauxie. The ancestor and the SS ecotype accumulated acetate as they depleted glucose (Anc: *t* = 5.418, *p* = 0.003; SS: *t* = 11.342, *p* = 0.0002); however, the FS strain did not accumulate acetate during glucose depletion, indicating that acetate is consumed during growth on glucose (*t* = 2.769, *p* = 0.025 not significant at experimentwise α = 0.017). For panels B and C, data are averages ± 1 mean standard error for three replicates, except for a single preinoculation replicate (open square).

The two ecotypes have repeatedly evolved from a single common ancestor, and their coexistence is maintained by negative frequency dependence generated by the daily, sequential depletion of resources [14–16]. This frequency dependence is likely to be generated by a trade-off between the metabolism of glucose and acetate [14]: a short switching time from glucose to acetate consumption is possible if acetate metabolism is active even during growth on glucose, but this in turn reduces the efficiency of glucose metabolism [17]. Because growth rate differences better reflect the selection pressures that caused the bacteria to diversify, we will refer to “slow-switchers” (SS), which correspond to Large colonies, and “fast-switchers” (FS), which refers to Small colonies.

A fundamental advantage of the E. coli model over evolution in natural systems is the ability of E. coli to survive cryogenic preservation. Ecological and phenotypic change in divergent E. coli strains that evolved from a common clone can be compared to the cryogenically preserved and revived common ancestor, and the ancestral strain can be manipulated genetically to contain alleles from the derived ecotypes and vice versa. These advantages have allowed us to gain an integrative understanding of genetic, phenotypic, and ecological mechanisms underlying sympatric adaptive diversification due to competition for resources in E. coli. Here, we analyse the changes in resource consumption and the concomitant genetic changes of a lab-based, well-documented evolutionary diversification.

## Results/Discussion

We isolated FS and SS strains from a diversified bacterial population based on their growth profiles and confirmed that the FS switching lag, i.e., the time elapsed between the end of growth on glucose and the maximum growth rate on acetate, was shorter than that of the ancestral and the SS strains (*F_2,12_* = 2841.6, *p* < 0.0001, Figure 1A). To test the hypothesis that only the FS type has an active acetate metabolism during growth on glucose, we measured the dynamics of acetate concentrations for the different strains over a full day's growth. In wild-type E. coli, acetate metabolism is repressed during growth on glucose [18], which incidentally generates acetate as a by-product. In wild-type strains, the net concentration of acetate in the medium should therefore increase during growth on glucose. For randomly selected strains of the FS and SS ecotypes and of their common ancestor, we monitored how the glucose and acetate concentrations changed during log growth on glucose (hours 0 to 4 in Figure 1A). As was expected if acetate metabolism is inactivated by the presence of glucose, both the ancestor and the SS generated acetate as a by-product during the glucose consumption phase of growth, so that acetate concentration in the medium increased (Figure 1B and 1C). In a striking deviation from this pattern, the FS strain did not accumulate acetate in its medium as it consumed glucose (Figure 1B and 1C). This indicated probable failure of a genetic mechanism to repress acetate usage during growth on glucose. It is likely that failed repression of acetate metabolism in turn allows for a fast diauxic shift to acetate consumption at the end of the glucose phase.

In an attempt to test this, we investigated the genetic basis of the de-repression of acetate metabolism in the FS strain. Likely acetate usage candidate genes include *acetyl-CoA synthetase (acs),* which converts acetate to acetyl-CoA, and the three-locus acetate operon, *aceBAK,* which converts acetyl-CoA derivatives to malate (Figure 2) [20]. The operon contains genes encoding malate synthetase A *(aceB),* isocitrate lyase *(aceA),* and isocitrate dehydrogenase kinase/phosphatase *(aceK),* all of which are coexpressed; we selected *aceB* as a proxy to represent expression of the operon.

**Figure 2:**
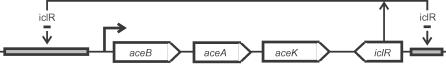
Schematic of Negative Regulation of the Acetate Operon *aceBAK* and the *iclR* Gene by iclR [22,33] Failure to negatively regulate *aceBAK* would result in constitutive expression of the operon.

We measured expression levels using quantitative PCR of *aceB* for the ancestral and derived strains in medium containing acetate as the sole carbon source, and contrasted this with expression in media containing glucose. When grown on acetate alone, a*ceB* levels were high and equivalent for the ancestral, SS, and FS strains (Figure 3). This confirms the expectation that the acetate metabolism is active in all three types when growing on acetate. However, when growing on glucose, expression levels of the *aceB* gene dropped dramatically in the ancestor and the SS strain (Figure 3). This occurred independently of whether acetate was present in the medium or not and confirmed that, in these strains, acetate metabolism is repressed during growth on glucose. In contrast, the FS strain continued to express high levels of *aceB,* and by inference the entire acetate operon, even when growing on glucose (Figure 3). In the glucose-containing media, *aceB* expression in the FS was reduced relative to the expression levels in acetate-only medium, but remained high relative to the SS and ancestral expression. The high *aceB* expression levels of the FS during growth on glucose strongly indicate that the FS ecotype has evolved a genetic mechanism by which the acetate operon remains expressed in the presence of glucose.

**Figure 3:**
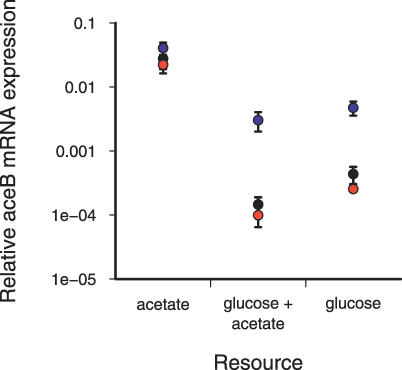
Relative mRNA Expression from the *aceB* Gene *aceB* mRNA expression was quantified using quantitative PCR and normalized against *23SrRNA-4* ribosomal mRNA expression. RNA was isolated from cells in early log growth during the first phase of diauxie when glucose was present. In glucose-containing media, the FS has significantly higher *aceB* expression than the Anc and SS (*F_8,18_* = 16.26, *p* < 0.0001; Tukey HSD_FS,SS_ *p* = 0.032). Symbols: Anc (black), SS (red), and FS (blue).

The enhanced *aceB* expression in the FS ecotype has two potential genetic causes: a change in the regulatory sequence of the acetate operon or a mutation in one of the operon's regulators. Through sequencing we confirmed that the regulatory region of *aceB* is identical in the FS, ancestor, and the SS. Next, we looked for mutations in the negative regulators of *aceBAK* because a decrease in negative regulation would cause constitutive expression of the *aceBAK* operon, congruent with the observed expression level changes. For the FS strain, we discovered that the isocitrate lyase repressor *(iclR)* gene, a negative regulator of *aceBAK* [21,22], contains a transposable *IS1* genetic element that terminates the *iclR* transcript when it is two-thirds complete. To determine whether this mutation was prevalent in the FS population in addition to our focal FS strain, we screened nine subsequent isolated FS and SS strains for this *iclR^IS1^* allele: eight of nine FS strains carried the mutant allele, but neither of the SS strains, nor the ancestor, carried this insertion. We additionally PCR screened FS genotypes from two similarly evolved populations and recovered only alleles of ancestral size at this locus. Indeed, we would not expect the exact same mutation (i.e., insertion of a transposon) to occur at the same site in two independently evolved populations, as it is likely that there are many different genetic mechanisms by which regulation of acetate metabolism can be altered.

To determine how the *IS1* insertion in the *iclR* gene affected acetate use in the FS, we substituted the ancestral *iclR^Anc^* allele into the FS genetic background and then estimated growth profile characteristics of the genetically modified strain. Inserting the ancestral *iclR* allele resulted in FS strains that had a significantly longer lag when switching from glucose to acetate use (Figure 4A and 4C), however this altered lag was still significantly shorter than that of the ancestral strain (Figure 4C). We concluded from this that the mutant *iclR^IS1^* allele in the FS ecotype decreases the amount of time the FS requires to switch from consuming glucose to metabolising acetate, presumably because this allele deregulates the acetate operon and thereby enhances acetate metabolic activity during growth on glucose.

**Figure 4:**
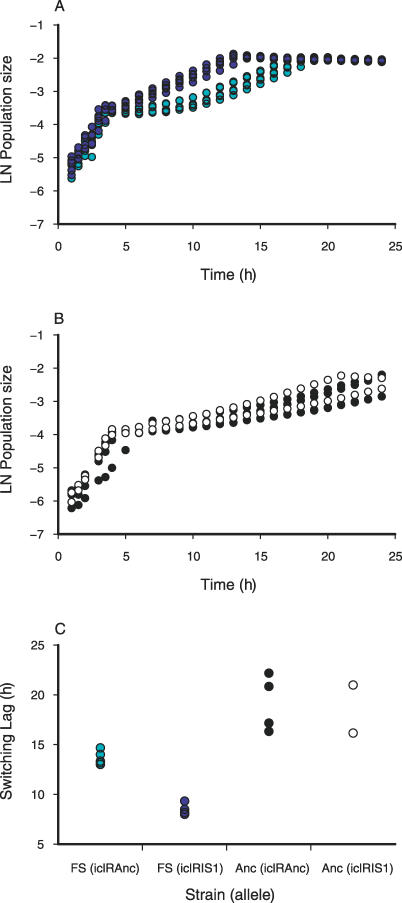
Growth Curves and Switching Lags for Genetically Derived Strains The *iclR^IS1^* allele (dark blue) alters switching lag relative to the *iclR^Anc^* allele (light blue) in the genetic background of the FS ecotype (A and C) but not the ancestral genetic background with alleles shown in white and black, respectively (B and C; *F_3,12_* = 32.0, *p* < 0.0001). Growth rates on glucose and acetate are not measurably affected by alleles at this locus (unpublished data; glucose: *F_3,12_* = 28.0, *p* < 0.0001; acetate: *F_3,11_* = 157.5, *p* < 0.0001).

We also inserted the FS mutant *iclR^IS1^* allele into the ancestral genetic background, although recombinants were much less common in this direction, a fact that provides some evidence for genetic interactions between this locus and genes in the ancestral genome. In these strains with the ancestral genetic background, the mutant *iclR^IS1^* allele did not affect switching times, which remained long (Figure 4B and 4C). We speculate that the rarity of allelic recombinants during the allelic replacement procedure and the lack of change in switching lag indicate the presence of epistatic effects. In particular, the *iclR^IS1^* insertion only seems to be effective in the genetic background of derived strains, but not in the genetic background of ancestral strains. This would not really be surprising, as the ancestor has no known evolutionary history in the glucose-acetate resource environment and does not carry the set of adaptively beneficial mutations that FS and SS must carry at other loci, based on differences in their growth curves from the ancestor (Figure 1A). We hypothesize that one or more derived alleles interact with the *iclR* locus to compound the effect of the *iclR^IS1^* insertion in the derived strains.

Although the *IS1* element disrupts the down-regulation of the acetate operon sufficiently to alter the switching time between resources, it does not exert sole control over the switching lag in the FS. This is evident because the FS with the *iclR^Anc^* allele switched to acetate earlier than either of the genetically modified ancestral strains (Figure 4C). Furthermore, in the genetically modified strains, the *iclR^IS1^* (FS) allele did not significantly affect colony morphology or growth rates on glucose or acetate (unpublished data). This clearly indicates that this single mutation is not sufficient to cause all of the resource use changes between FS and the ancestor, or between FS and SS. In particular, *iclR* does not act alone to cause the critical trade-off in performance that enables coexistence between the FS and SS strains. (Note that the FS strain and the ancestor never coexisted in the same population, and hence we would not expect to see evidence of a trade-off between these strains.) The observed differences in the growth rates and the colony morphology between the various strains must therefore result from additional modifications to metabolism. Nevertheless, our data show that a genetic change in the regulation of genes controlling carbohydrate metabolism has contributed substantially to the differentiation of coexisting ecotypes in E. coli populations.

Our results not only confirm that regulatory changes can provide a mechanism for rapid evolutionary change [4,6–8,23], but they show that such regulatory changes may play a crucial role in processes of sympatric diversification. The results also show that such regulatory changes can act upon phylogenetically ancient central metabolic pathways such as the acetate switch found in microorganisms as diverse as gram negative E. coli, gram-positive Bacillus subtilis, and halophilic archea Haloferax volcanii [24]. The importance of acetate utilization on niche adaptation in nature is evident within the gammaproteobacteria, the taxonomic class containing E. coli, as evolutionary changes in the acetate utilization are correlated with pathogenicity in both closely related species such as *Shigella* [25] and less closely related species such as *Yersinia* spp [26]. Our current results demonstrate that similar evolutionary changes can also be observed in the laboratory during experimental evolution, and that such regulatory changes are important in niche specialization and differentiation [8]. These results establish a link between different levels of biological organization by showing how a genetic modification of gene regulation affects the expression of genes that are important for metabolic pathways, and how this gene expression in turn affects a trade-off in resource use that causes disruptive selection and competitive diversification.

## Materials and Methods

### Strains and general culture.

Previously, we conducted an evolution experiment in which Populations 29, 31, and 33 were derived after 100 generations of evolution from an E. coli B strain (REL606 [27], hereafter called Anc for ancestor) under a regimen of daily batch culture in Davis minimal medium supplemented with glucose and acetate [14,15]. Strains 33A (SS) and 33K (FS) were isolated from generation 1,000 (day 150) of population 33 and have the characteristic growth rate parameters (see growth curve assay below) and colony morphologies for their respective ecotypes. SS colonies grow within 24 h on tryptone agar plates containing 0.005% tetrazolium dye, whereas FS colonies require >24 h to grow and are visibly smaller than SS colonies after 48 h. All assays requiring liquid cultures were conducted in Davis minimal (DM) medium and supplemented with 0.0002% thiamine HCl, 0.1% MgSO_4_ [28], and carbon sources as follows: 410 mg/L D-glucose monohydrate (DM-glucose), 410 mg/L sodium acetate trihydrate (DM-acetate), 205 mg/L D-glucose monohydrate, and 205 mg/L sodium acetate trihydrate (DM50:50), unless otherwise noted. All bacteria were cultured at 37 °C. All liquid cultures were aerated by shaking at 250 rpm unless otherwise indicated.

### Glucose consumption and acetate production.

Three independent cultures of each of FS, SS, and Anc were sampled throughout log growth in DM50:50 to determine glucose and acetate concentrations. For each sample, we removed approximately 1 ml of culture, centrifuged to pellet the cells, and stored the supernatant at −80 °C. After all samples were obtained, we estimated the glucose concentrations in the supernatant using a Glucose (HK) Assay kit (Sigma, http://www.sigmaaldrich.com), and acetate concentrations were determined using a UV-method Acetic Acid kit (Boehringer Mannheim/R-biopharm, http://www.r-biopharm.com). Manufacturer-supplied standards and non-inoculated media samples were used as controls.

### PCR.

Colonies were PCR assayed for the presence of the *IclR^IS1^* allele. The sample included nine FS and ten SS from population 33 and three FS and three SS from each of two other populations (29 and 31) evolved under identical conditions. PCR was conducted in 25 μl volumes (400 nM each primer, 400 μM dNTPs, 1X reaction buffer, and 5 U Taq [Roche, www.roche-diagnostics.com]). Primers 5′-TCGAAAATACACGCTGCAAG and 5′-TTCCACTTTGCTGCTCACAC amplified from 217 bp upstream of the *IclR* start site through 83% of the gene. The template for each reaction was sampled from a single colony grown for 24 h (for SS and Anc) or 48 h (for FS). Reaction conditions were as follows: 95 °C for 10 min, 30 cycles amplification (95 °C for 30 s, 64 °C for 30 s, and 72 °C for 1 min), and final extension period (72 °C for 5 min). PCR products were size separated in 2.0% agarose to assay for the slower migration of the *iclR^IS1^* allele relative to the wild type.

### Sequencing.

For the regulatory regions of *acs*, *aceBAK*, and *iclR* and for the *iclR* gene, we sequenced strains 33A, 33K, and Anc. Additional sequence data were collected for *acs* from one additional FS and SS from population 33 as well as two FS and two SS from two additional populations. Sequencing was performed by the University of British Columbia's Nucleic Acid and Protein Service (NAPS), using the ABI PRISM Big Dye V.3.1 sequencing kit (Applied Biosystems, http://www.appliedbiosystems.com). Primers were from Treves et al*.* [29] (*acs* regulatory region), 5′-GCTGGCGTAGTCACGGTAAT and 5′-ATCGGTTGTTGTTGCCTGTT (*aceB* regulatory region), or as for PCR and qPCR.

### Quantitative PCR.

Three independent samples each of 33K, 33A, and Anc were prepared from early log growth in DM-glucose, DM-acetate, and DM50:50. Cultures were collected on ice, pelleted, and resuspended in RNAlater (Ambion, http://www.ambion.com). RNAs were prepared using RNeasy (Qiagen, http://www.qiagen.com) or Ribopure (Ambion) kits, following the manufacturers' protocols. Remaining genomic DNA was removed using DNAfree (Ambion). cDNAs were generated using the TaqMan kit (Applied Biosystems), primed with random hexamers.

Reactions were carried out in duplicate in a 7000 SDS (ABI) using SYBR green master mix (ABI) according to the manufacturers protocol and primers designed with Primer Express 2.0 (ABI). Primers used were: 5′-TGGCGTGGTGAGGCAAT and 5′-GGAAGAAATAGAGCGCAAAATCA *(aceB)* and 5′-GCTGATACCGCCCAAGAGTTC and 5′-CAGGATGTGATGAGCCGAC *(23SrRNA-4).* Controls lacking reverse transcriptase were included for each sample to detect genomic DNA contamination and primer dimerization. As well, dissociation curves were used to confirm a single product. Standard curves were prepared for each primer set using five serial 5-fold dilutions of Anc genomic DNA template.

RNA abundance was quantified by normalizing the quantity of cDNA *aceB* template to the quantity of cDNA *23SrRNA-4* template, chosen as a control because of its unchanging, high expression in both glucose and acetate growth conditions [20].

### Allelic replacement.

We transferred *iclR^IS1^* to Anc and *iclR^Anc^* into FS using a suicide plasmid mediated technique [30] but substituting the suicide plasmid pRE112 [31]. Control transfers to return each allele into its original genetic background were also performed.

### Growth curve assay.

Growth curve data were generated using a Bioscreen C plate reader (MTX Lab Systems Inc, http://www.mtxlsi.com). Bacteria were inoculated into assay wells containing 250 μl aliquots of DM supplemented with 0.0002% thiamine HCl, 0.1% MgSO_4_, and one of three resource combinations: 410 mg/L glucose, 410 mg/L acetate, or 41 mg/L glucose and 369 mg/L acetate (DM10:90). Cultures were grown for 24 h at 37 °C with continuous shaking and measured for wide band optical density every 10 min. Five technical replicates of each sample were averaged after anomalous growth curves were removed. We used C++ code provided by J. Tyerman to estimate three growth curve parameters: the maximum growth rates on glucose and acetate and switching time between resources, defined as the time elapsed between the end of growth on glucose and the maximum growth rate on acetate. Briefly, the code linearizes the data over a sliding window of nine time units to establish the changes in slope of the optical density over a 24-h growth period. The first critical point where the slope changes from positive to zero (or negative) defines the switching point. We calculate the glucose growth rate as the maximum slope between time 0 and the switching point, the acetate growth rate as the maximum slope between the switching point and the time of maximum optical density, and the switching lag as the difference in time units between the switching point and the maximum growth rate on acetate. Analyses of variance (ANOVAs) were calculated for these three parameter estimates for the FS, SS, and Anc strains and for the four genetically modified strains, Anc*(iclR^Anc^),* Anc*(iclR^IS1^),* FS*(iclR^Anc^),* and FS*(iclR^IS1^),* described above. ANOVAs were conducted in JMP [32] using Tukey Honestly Significant Difference (HSD) multiple comparisons. All Tukey HSD tests were significant for the FS, SS, and Anc multiple comparisons. Results from the genetically modified strains are given in Figure 4.

## Supporting Information

### Accession Numbers

The EchoBASE (http://www.biolws1.york.ac.uk/echobase) accession numbers for the E. coli strains discussed in this paper are acetyl-CoA synthetase (EB1417), malate synthetase A (EB0022), isocitrate lyase (EB0021), isocitrate dehydrogenase kinase/phosphatase (EB0025), and isocitrate lyase repressor (EB0486).

## Abbreviations

Anc: ancestral strain
SS: slow-switchers
FS: fast-switchers
